# The prognostic value of programmed death-ligand 1 (PD-L1) expression in resected colorectal cancer without neoadjuvant therapy - differences between antibody clones and cell types

**DOI:** 10.1186/s12885-024-12812-7

**Published:** 2024-08-26

**Authors:** Hampus Nobin, Stina Garvin, Helga Hagman, Björn Nodin, Karin Jirström, Hans Brunnström

**Affiliations:** 1 Department of Pathology, Region Kalmar, Kalmar County Hospital, Kalmar, Sweden; 2 Department of Clinical Sciences Lund, Division of Pathology, Lund University, Lund, Sweden; 3 Department of Clinical Pathology, Department of Biomedical and Clinical Sciences, Linköping University, Linköping, Sweden; 4 Department of Clinical Sciences Lund, Division of Oncology and Therapeutic Pathology, Lund University, Lund, Sweden; 5 Department of Genetics, Pathology, and Molecular Diagnostics, Regional University Laboratories, Skåne University Hospital, Lund, Sweden

**Keywords:** 22C3, 73-10, Immune cells, SP263, Survival

## Abstract

**Background:**

Programmed death-ligand 1 (PD-L1) expression on tumor cells is associated with poor prognosis in several malignancies, while partly contradictory and inconclusive results have been presented for colorectal cancer (CRC). This study aimed to evaluate PD-L1 as a prognostic biomarker in CRC by comparing three different antibody clones.

**Methods:**

Patients surgically treated for CRC between January 1st, 2007, and December 31st, 2015, in Kalmar County, Sweden, were retrospectively included. Tissue microarrays from 862 primary tumors without neoadjuvant treatment were assessed for immunohistochemical expression of PD-L1 in tumor cells (TC) and immune cells (IC) using clones 73-10, SP263, and 22C3. Cox regression proportional hazard models were used to estimate hazard ratios for overall survival (OS) and disease-free interval (DFI) in univariable and multivariable analyses, with 1% and 5% set as cut-offs for positive expression in TC and IC respectively.

**Results:**

PD-L1 expression in TC was found in 89 (10%) cases for clone 73-10, 76 (9%) for clone SP263, and 38 (4%) for clone 22C3, while the numbers for IC were 317 (37%) cases for clone 73-10, 264 (31%) for clone SP263, and 89 (10%) for clone 22C3. PD-L1 expression in IC was associated with prolonged OS and DFI in univariable analysis for all three clones. The link to prolonged DFI remained in multivariable analysis for 73-10 and SP263, but only for 73-10 regarding OS. PD-L1 expression in TC was not prognostic of OS in any analysis, while it was associated with prolonged DFI for SP263, and a trend was seen for 73-10. The link to prolonged DFI remained for SP263 and was strengthened for 73-10 in multivariable analysis.

**Conclusions:**

The prognostic value of PD-L1 expression in both IC and TC differs between antibody clones, with 73-10 and SP263 being more reliable for prognostic information than 22C3 in resected CRC.

**Supplementary Information:**

The online version contains supplementary material available at 10.1186/s12885-024-12812-7.

## Background

Colorectal cancer (CRC) is the second most common cancer in women and the third most common cancer in men worldwide. Despite improved treatment strategies, CRC remains the second leading cause of cancer-related deaths. In 2020, over 1.9 million people were newly diagnosed with CRC and about 935,000 people died from CRC [1].

Currently, surgical resection with the addition of chemotherapy in advanced cases is the mainstay of CRC treatment. The use of immune checkpoint inhibition (ICI), monoclonal antibodies blocking programmed death receptor-1 (PD-1), programmed cell death-ligand 1 (PD-L1), or cytotoxic T-lymphocyte associated protein 4 (CTLA-4), has lately become the standard of care for several malignancies such as melanoma, non-small cell lung cancer, and renal cell carcinoma [2, 3]. Treatment with ICI is also approved as first- and second-line treatment for advanced CRC with deficient mismatch repair (dMMR) [4, 5]. Promising results have been reported for neoadjuvant treatment in early-stage dMMR CRC [6, 7].

Tumor-infiltrating lymphocytes (TILs) are widely considered to reflect the primary host immune response against solid tumors. In CRC tissue, increased TILs are associated with improved outcomes as well as with dMMR [8, 9]. However, the immune system is equipped with several inhibitory mechanisms to prevent excessive lymphocyte activation [10]. PD-L1, usually expressed by macrophages, some activated T cells and B cells, dendritic cells, and some epithelial cells, is an immune-regulatory molecule that, upon interacting with its receptor, PD-1, expressed by both lymphoid and non-lymphoid immune cells, suppresses the CD8 cytotoxic immune response in both physiologic and pathologic pathways [11, 12]. PD-L1 expression by cancer cells or different types of host cells in the tumor microenvironment leads to downregulation of the immune system followed by immune escape [13].

PD-L1 expression on tumor cells (TC) has been shown to contribute to an impaired host immune response and subsequent poor prognosis in several malignancies [14–16]. Regarding the expression of PD-L1 and its prognostic value in CRC, the results are contradictory. Although many studies indicate that PD-L1 expression is associated with a worse prognosis [17–21] others report no prognostic impact [22]. Some studies even suggest that PD-L1 expression is associated with a better prognosis [10, 23]. Also, based on data from The Cancer Genome Atlas (TCGA) and the Human Protein Atlas (HPA), high expression of PD-L1 at the mRNA level is shown to correlate with favourable prognosis and prolonged survival for patients with CRC [24]. However, the studies differ in design and study populations. Currently, there is no standardized way to assess PD-L1 expression in CRC, thus the investigated cell population, the choice of antibody clones, and the scoring methods vary between studies.

The aim of the present study was to evaluate different PD-L1 clones regarding potential differences in expression and prognostic information in a well-defined cohort of patients with surgically resected CRC without prior neoadjuvant therapy.

## Methods

### Study design, patients, and data collection

In this retrospective cohort study from Kalmar County, Sweden, with an uptake area of approximately 230,000 individuals, all patients surgically treated for CRC between the 1st of January 2007 and the 31st of December 2015 were eligible. Patients were identified by a search of the laboratory information system at the Department of Pathology in Kalmar County (*n* = 1217). Exclusion criteria were insufficient tumor material (*n* = 59), death within 28 days of surgery (*n* = 49), treatment with neoadjuvant chemotherapy/radiotherapy (*n* = 206), no possibility of follow-up (*n* = 11), and more than one CRC at the time of surgery (*n* = 7). In 23 of the included cases, there was not enough tumor tissue in the tissue microarrays (TMA) for evaluation. The final study population comprised 862 patients (see Supplementary Fig. 1 for a flowchart). Hematoxylin and eosin-stained sections of all formalin-fixed paraffin-embedded (FFPE) tissue blocks were reviewed by a senior pathologist (HN) to confirm the presence of adequate tumor content for TMA construction and to evaluate pathological features including histologic tumor type, differentiation grade, TNM stage, extramural vascular invasion (EMVI), perineural invasion (PNI), perforation, circumferential resection margin (CRM), tumor deposit, and tumor budding. TNM staging was performed in accordance with UICC TNM Classification of Malignant Tumors, 8th ed. Clinical and treatment data such as age, sex, primary site, neoadjuvant and/or adjuvant treatment, disease-free interval (DFI), and overall survival (OS) were obtained from pathology records and medical charts. Neoadjuvant and/or adjuvant treatment was defined as treatment longer than one month. OS was defined as the time from surgical treatment to death from any cause. DFI was defined as the time from surgical treatment to the first recurrence of CRC or death from the CRC disease. Patients with up-front metastatic disease (stage IV), where all metastases could not be radically removed, were excluded from the DFI analysis (*n* = 61). In this retrospective cohort with a long follow-up time, we chose to use DFI, as it better reflects the tumor biology and the risk of recurrence than does disease-free survival (DFS), which does not distinguish between death from CRC or death from any cause. Patients alive at the end of follow-up, patients who died from other causes, or were diagnosed with another cancer during follow-up were censored from the date of the event. Patients were followed from the date of CRC diagnosis to death or until September 23rd, 2023, whichever came first. The study was approved by the Ethics Committee of Linköping (ref nr 2018/342-31).

### Tissue microarray construction

FFPE tissue blocks from all the included cases were obtained from the Department of Pathology in Kalmar. Representative and non-necrotic areas were marked on corresponding hematoxylin and eosin-stained slides, and TMAs were constructed with duplicate tissue cores (1 mm) taken from each primary tumor and mounted in recipient blocks, using a semi-automated arraying device (TMArrayer, Pathology Devices, Westminster, MD, USA). From these recipient blocks, 4 μm thick sections were subsequently cut, using a microtome, and mounted on glass slides.

### Immunohistochemistry

For immunohistochemical (IHC) analysis of PD-L1 antibody clone SP263 (ready to use, Ventana) and antibody clone 22C3 (1:40, pharmDX) 4 μm thick TMA-sections were pretreated with ULTRA Cell Conditioning Solution 1 (CC1). Staining was performed using a Ventana BenchMark ULTRA (Ventana Medical System, Tucson, Arizona, USA) automated IHC slide staining system. The antibodies were visualized using the OptiView DAB IHC Detection Kit (no. 760-700, Ventana). For IHC analysis of PD-L1 antibody clone 73-10 (1:500, Abcam) 4 μm thick TMA-sections were pretreated with FLEX TRS High, PT Link system and then stained using an Autostainer Plus (Dako, Glostrup, Denmark). The antibody was visualized using the EnVision DAB Detection System (Agilent/Dako). Tonsil tissue was included as a positive control for low expression (macrophages in germinal centers) and high expression (crypt epithelium) on all slides.

Screening for microsatellite instability (MSI) was performed using IHC. Tumors were considered dMMR when expression (nuclear staining) of one or more of the mismatch repair proteins (MLH1, PMS2, MSH2, and MSH6) was lost. Tumors with intact mismatch repair proteins were considered MMR proficient (pMMR). Antibodies against MLH1 (clone M1, ready to use, Ventana), PMS2 (clone A16-4, ready to use, Ventana), MSH2 (clone G219-129, ready to use, Ventana), and MSH6 (clone SP93, ready to use, Ventana) were used. Pretreatment, staining, and visualization were performed in the same manner as for antibodies SP263 and 22C3.

### Scoring of PD-L1 expression

IHC PD-L1 expression was evaluated based on linear membranous staining of the TC and any staining of the immune cells (IC; lymphocytes and macrophages) infiltrating the tumor and/or tumor stroma. The estimated percentage of stained TC and IC respectively were scored semi-quantitatively as 0 (< 1%), 1 (1–4%), 2 (5–9%), 3 (10–49%), and 4 (50–100%). TC were scored based on any cell membrane staining, partial or complete, regardless of intensity. For IC, both membranous and cytoplasmatic staining were considered, regardless of intensity. All stainings were evaluated by one senior pathologist (HN). Data were dichotomized for prognostic evaluation using 1% PD-L1 expression in TC and 5% in IC as cut-offs. In the absence of standardized grading systems, cut-offs were based on previous studies and on differences in survival analyses (Kaplan-Meier curves).

### Statistics

Statistical analysis was performed using Statistica (Version 13.5.0.17, TIBCO Software Inc) and MedCalc (version 14.12.0, MedCalc Software bvba, Ostend, Belgium). All statistical tests were two-sided and p-values < 0.05 were considered statistically significant. Pearson’s chi-squared tests were used to evaluate associations between PD-L1 expression and clinicopathological variables. Survival curves were generated according to the Kaplan-Meier method and the log-rank test to investigate differences in DFI and OS according to PD-L1 expression. Cox regression proportional hazard models were used to estimate hazard ratios (HRs) for OS and DFI in both univariable and multivariable analyses. Variables with p-value < 0.1 in univariable analysis were considered in the multivariable model to investigate the presence of any independent prognostic value.

## Results

A total of 862 patients were included in the study cohort. The median age at the time of surgery was 74 years (range 33–97), and 52% were females. The mean and median follow-up time was 7.3 and 7.8 years respectively (range 1.4-202.2 months). The shortest follow-up time was 94.5 months for patients alive at the follow-up closure date. During follow-up 552 (64%) patients died, 197 (23%) experienced disease recurrence, and 54 (6%) were diagnosed with another cancer. The clinicopathologic characteristics of the study cohort are shown in Table 1.

**Table 1:** Clinicopathologic characteristics in the whole cohort of 862 resected colorectal cancers without neoadjuvant treatment

	Cohort, *n* (%)		Cohort, *n* (%)
**Cases**	862 (100)	**Mucinous histology**	
**Age**		** Absent**	687 (80)
**<74**	376 (44)	** Present**	175 (20)
**≥74**	486 (56)	**CRM**	
**Sex**		** >0 mm**	825 (96)
**Male**	414 (48)	** 0 mm**	37 (4)
**Female**	448 (52)	**MMR**	
**Site**		** pMMR**	672 (78)
**Right Colon**	468 (54)	** dMMR**	190 (22)
**Left Colon**	289 (34)	**EMVI**	
**Rectum**	105 (12)	** No**	534 (62)
**T stage**		** Yes**	328 (38)
**1**	23 (3)	**PNI**	
**2**	88 (10)	** No**	758 (88)
**3**	611 (71)	** Yes**	104 (12)
**4**	140 (16)	**Tumor budding**	
**N stage**		** Bd1**	462 (54)
**0**	478 (55)	** Bd2**	239 (28)
**1**	217 (25)	** Bd3**	161 (19)
**2**	167 (19)	**Tumour deposits**	
**M stage**		** No**	791 (92)
**0**	775 (90)	** Yes**	71 (8)
**1**	87 (10)	**Perforation**	
**Stage**		** No**	838 (97)
**I**	96 (11)	** Yes**	24 (3)
**II**	367 (43)	**EO**	
**III**	312 (36)	** No**	828 (96)
**IV**	87 (10)	** Yes**	34 (4)
**Tumor grade**		**Adjuvant treatment**	
**Low grade**	632 (73)	** No**	660 (77)
**High grade**	230 (27)	** Yes**	202 (23)

*Abbreviations* CRM, circumferential resection margin; dMMR, deficient mismatch repair; EMVI, extramural vascular invasion; EO, emergency operation; MMR, mismatch repair; pMMR, proficient mismatch repair; PNI, perineural invasion

**Fig. 1:**
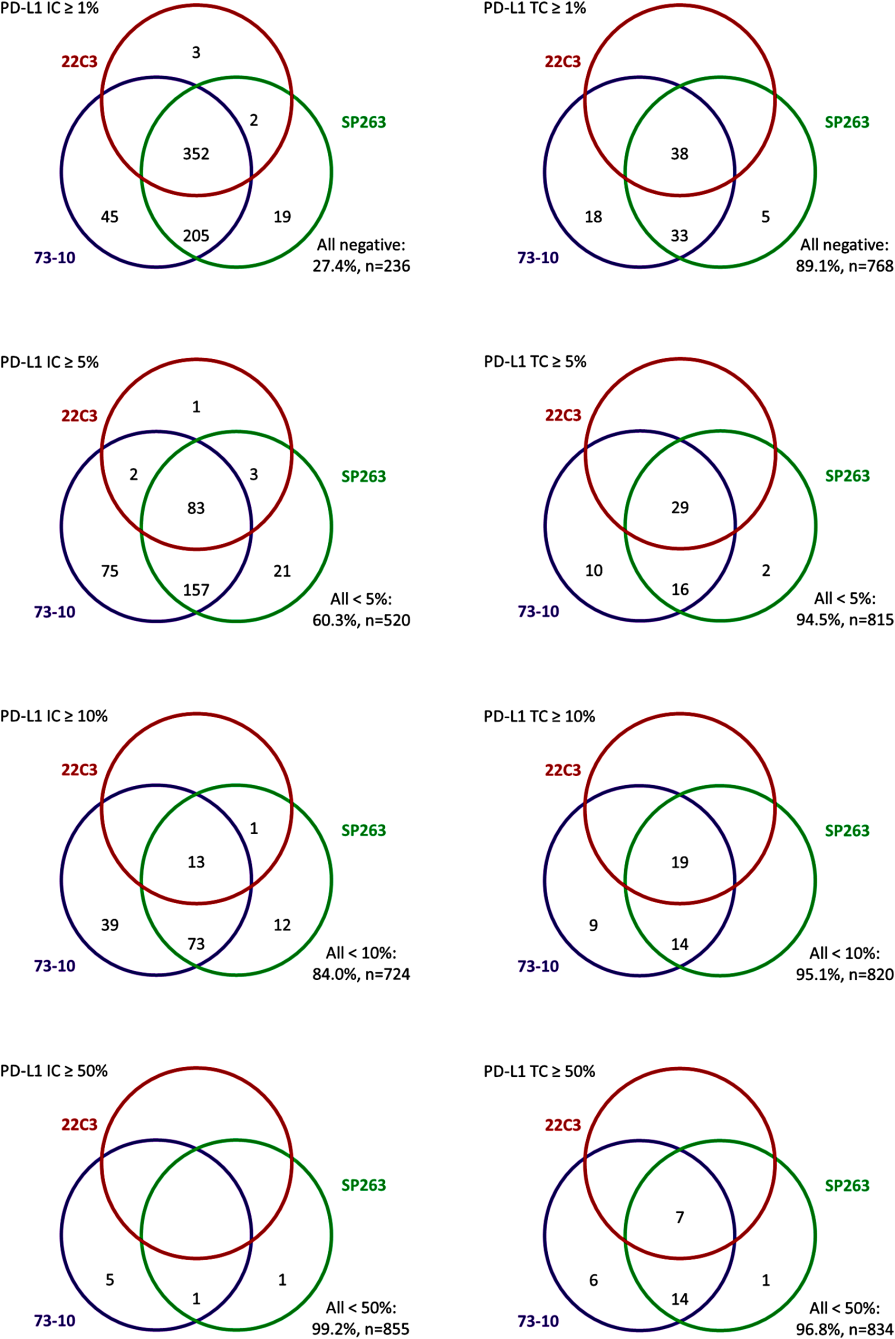
Venn diagram showing the concordance of programmed death ligand-1 (PD-L1) expression in tumor cells (TC) and immune cells (IC) for different antibody clones at various cut-offs in 862 cases of non-neoadjuvant treated resected colorectal cancer

PD-L1 expression in TC (cut-off 1%) was found in 89 (10%) cases for clone 73-10, 76 (9%) cases for clone SP263, and 38 (4%) cases for clone 22C3. PD-L1 expression in IC (cut-off 5%) was found in 317 (37%) cases for clone 73-10, 264 (31%) cases for clone SP263, and 89 (10%) cases for clone 22C3. Thirty-eight cases (4%) were identified as PD-L1 positive in TC by all three clones and 83 (10%) cases were identified as PD-L1 positive in IC by all three clones. The concordances between the different clones at various cut-offs are presented as Venn diagrams in Fig. 1. The prevalences of PD-L1 expression for the different clones (73-10, SP263, and 22C3) at various cut-offs are presented in Supplementary Table 1.

To investigate possible differences in PD-L1 expression depending on the age of the tissue, the cohort was divided into three time periods (2007 through 2009, 2010 through 2012, and 2013 through 2015). However, obvious differences in the expression of PD-L1 depending on tissue age could not be demonstrated for any antibody clone. In essence, positivity was slightly more frequent for TC and slightly less frequent for IC in older blocks for all clones. Results are presented in Supplementary Table 2.

As evident from Table 2, PD-L1 expression in TC was related to female gender, right-sided primary tumor location (PTL), high tumor grade, absence of mucinous histology, dMMR, and presence of tumor perforation for all three clones. Further, PD-L1 expression was related to high age (≥ 74 years) for clones 73-10 and 22C3. None of the other clinicopathological parameters showed any relation to PD-L1 expression in TC. PD-L1 expression in IC was related to right-sided PTL, lower T stage, lower N stage, no distant metastasis (M0), lower total stage, dMMR, absence of EMVI, low extent of tumor budding, and absence of tumor deposits for all three clones. PD-L1 expression in IC was also related to female gender and low tumor grade for clone 73-10. Further, the absence of mucinous histology was related to PD-L1 expression in IC for SP263 and 22C3, while the absence of PNI and no administration of adjuvant therapy was related to PD-L1 expression in IC for 73-10 and SP263. Finally, PD-L1 expression in TC was linked to PD-L1 expression in IC for all clones. Details on the clinicopathologic characteristics of the study cohort and their associations with PD-L1 expression are shown in Table 2.

**Table 2:** Clinicopathologic characteristics and correlations to immunohistochemical PD-L1 expression for three different antibody clones in resected colorectal cancers without neoadjuvant treatment

	PD-L1 clone 73-10, *n* (%)						PD-L1 clone SP263, *n* (%)						PD-L1 clone 22C3, *n* (%)					
	**TC** **≥ 1%**	**TC < 1%**	**p-value**	**IC** **≥ 5%**	**IC** **< 5%**	**p-value**	**TC** **≥ 1%**	**TC < 1%**	**p-value**	**IC** **≥ 5%**	**IC** **< 5%**	*p*-value	**TC** **≥ 1%**	**TC < 1%**	*p*-value	**IC** **≥ 5%**	**IC** **< 5%**	*p*-value
**Cases**	89 (10)	773 (90)		317 (37)	545 (63)		76 (9)	786 (91)		264 (31)	598 (69)		38 (4)	824 (96)		89 (10)	773 (90)	
**Age**																		
**<74**	28 (31)	348 (45)	0.015	130 (41)	246 (45)	0.239	28 (37)	348 (44)	0.212	107 (41)	269 (45)	0.224	9 (24)	367 (45)	0.011	33 (37)	343 (44)	0.189
**≥74**	61 (69)	425 (55)		187 (59)	299 (55)		48 (63)	438 (56)		157 (59)	329 (55)		29 (76)	457 (55)		56 (63)	430 (56)	
**Sex**																		
**Male**	31 (35)	383 (50)	0.009	136 (43)	278 (51)	0.022	27 (36)	387 (49)	0.022	118 (45)	296 (49)	0.193	11 (29)	403 (49)	0.016	39 (44)	375 (49)	0.401
**Female**	58 (65)	390 (50)		181 (57)	267 (49)		49 (64)	399 (51)		146 (55)	302 (51)		27 (71)	421 (51)		50 (56)	398 (51)	
**Site**																		
**Right Colon**	74 (83)	394 (51)	< 0.001	193 (61)	275 (50)	0.001	63 (83)	405 (52)	< 0.001	160 (61)	308 (52)	< 0.001	35 (92)	433 (53)	< 0.001	54 (61)	414 (54)	< 0.001
**Left Colon**	13 (15)	276 (36)		81 (26)	208 (38)		10 (13)	279 (36)		59 (22)	230 (38)		2 (5)	287 (35)		11 (12)	278 (36)	
**Rectum**	2 (2)	103 (13)		43 (14)	62 (11)		3 (4)	102 (13)		45 (17)	60 (10)		1 (3)	104 (13)		24 (27)	81 (10)	
**T stage**																		
**1**	2 (2)	21 (3)	0.212	12 (4)	11 (2)	< 0.001	1 (1)	22 (3)	0.284	12 (5)	11 (2)	< 0.001	0 (0)	23 (3)	0.209	5 (6)	18 (2)	0.015
**2**	5 (6)	83 (11)		45 (14)	43 (8)		7 (9)	81 (10)		41 (16)	47 (8)		2 (5)	86 (10)		16 (18)	72 (9)	
**3**	62 (70)	549 (71)		228 (72)	383 (70)		50 (66)	561 (71)		183 (69)	428 (72)		26 (68)	585 (71)		56 (63)	555 (72)	
**4**	20 (22)	120 (15)		32 (10)	108 (20)		18 (24)	122 (16)		28 (11)	112 (19)		10 (26)	130 (16)		12 (13)	128 (17)	
**N stage**																		
**0**	44 (49)	434 (56)	0.089	212 (67)	266 (49)	< 0.001	39 (51)	439 (56)	0.589	182 (69)	296 (50)	< 0.001	17 (45)	461 (56)	0.361	63 (71)	415 (54)	0.004
**1**	20 (22)	197 (25)		63 (20)	154 (28)		19 (25)	198 (25)		50 (19)	167 (28)		11 (29)	206 (25)		11 (12)	206 (27)	
**2**	25 (28)	142 (18)		42 (13)	125 (23)		18 (24)	149 (19)		32 (12)	135 (23)		10 (26)	157 (19)		15 (17)	152 (20)	
**M stage**																		
**0**	85 (96)	690 (89)	0.064	305 (96)	470 (86)	< 0.001	71 (93)	704 (90)	0.287	253 (96)	522 (87)	< 0.001	36 (95)	739 (90)	0.312	87 (98)	688 (89)	0.009
**1**	4 (4)	83 (11)		12 (4)	75 (14)		5 (7)	82 (10)		11 (4)	76 (13)		2 (5)	85 (10)		2 (2)	85 (11)	
**Stage**																		
**I**	7 (8)	89 (12)	0.081	54 (17)	42 (8)	< 0.001	8 (11)	88 (11)	0.590	48 (18)	48 (8)	< 0.001	2 (5)	94 (11)	0.228	20 (22)	76 (10)	< 0.001
**II**	37 (42)	330 (43)		156 (49)	211 (39)		31 (41)	336 (43)		130 (49)	237 (40)		15 (39)	352 (43)		43 (48)	324 (42)	
**III**	41 (46)	271 (35)		95 (30)	217 (40)		32 (42)	280 (36)		75 (28)	237 (40)		19 (50)	293 (36)		24 (27)	288 (37)	
**IV**	4 (4)	83 (11)		12 (4)	75 (14)		5 (7)	82 (10)		11 (4)	76 (13)		2 (5)	85 (10)		2 (2)	85 (11)	
**Tumor grade**																		
**Low grade**	28 (31)	604 (78)	< 0.001	216 (68)	416 (76)	0.009	25 (33)	607 (77)	< 0.001	189 (72)	443 (74)	0.446	5 (13)	627 (76)	< 0.001	63 (71)	569 (74)	0.569
**High grade**	61 (69)	169 (22)		101 (32)	129 (24)		51 (67)	179 (23)		75 (28)	155 (26)		33 (87)	197 (24)		26 (29)	204 (26)	
**Mucinous histology**																		
**Absent**	82 (92)	605 (78)	0.002	261 (82)	426 (78)	0.142	72 (95)	615 (78)	0.001	222 (84)	465 (78)	0.033	37 (97)	650 (79)	0.006	80 (90)	607 (79)	0.012
**Present**	7 (8)	168 (22)		56 (18)	119 (22)		4 (5)	171 (22)		42 (16)	133 (22)		1 (3)	174 (21)		9 (10)	166 (21)	
**CRM**																		
**>0 mm**	82 (92)	743 (96)	0.079	309 (97)	516 (95)	0.051	70 (92)	755 (96)	0.105	255 (97)	570 (95)	0.395	34 (89)	791 (96)	0.052	85 (96)	740 (96)	0.921
**0 mm**	7 (8)	30 (4)		8 (3)	29 (5)		6 (8)	31 (4)		9 (3)	28 (5)		4 (11)	33 (4)		4 (4)	33 (4)	
**MMR**																		
**pMMR**	34 (38)	638 (83)	< 0.001	204 (64)	468 (86)	< 0.001	30 (39)	642 (82)	< 0.001	175 (66)	497 (83)	< 0.001	7 (18)	665 (81)	< 0.001	56 (63)	616 (80)	< 0.001
**dMMR**	55 (62)	135 (17)		113 (36)	77 (14)		46 (61)	144 (18)		89 (34)	101 (17)		31 (82)	159 (19)		33 (37)	157 (20)	
**EMVI**																		
**No**	63 (71)	471 (61)	0.069	229 (72)	305 (56)	< 0.001	54 (71)	480 (61)	0.087	193 (73)	341 (57)	< 0.001	29 (76)	505 (61)	0.062	70 (79)	464 (60)	< 0.001
**Yes**	26 (29)	302 (39)		88 (28)	240 (44)		22 (29)	306 (39)		71 (27)	257 (43)		9 (24)	319 (39)		19 (21)	308 (40)	
**PNI**																		
**No**	82 (92)	676 (87)	0.199	300 (95)	458 (84)	< 0.001	70 (92)	688 (88)	0.242	250 (95)	508 (85)	< 0.001	34 (89)	724 (88)	0.766	83 (93)	675 (87)	0.103
**Yes**	7 (8)	97 (13)		17 (5)	87 (16)		6 (8)	98 (12)		14 (5)	90 (15)		4 (11)	100 (12)		6 (7)	98 (13)	
**Tumor budding**																		
**Bd1**	47 (53)	415 (54)	0.987	201 (63)	261 (48)	< 0.001	39 (51)	423 (54)	0.871	174 (66)	288 (48)	< 0.001	24 (63)	438 (53)	0.474	59 (66)	403 (52)	0.032
**Bd2**	25 (28)	214 (28)		74 (23)	165 (30)		23 (30)	216 (27)		60 (23)	179 (30)		8 (21)	231 (28)		20 (22)	218 (28)	
**Bd3**	17 (19)	144 (19)		42 (13)	119 (22)		14 (18)	147 (19)		30 (11)	131 (22)		6 (16)	155 (19)		10 (11)	151 (20)	
**Tumour deposits**																		
**No**	85 (96)	706 (91)	0.175	303 (96)	488 (90)	0.002	73 (96)	718 (91)	0.154	254 (96)	537 (90)	0.002	38 (100)	753 (91)	0.059	87 (98)	704 (91)	0.029
**Yes**	4 (4)	67 (9)		14 (4)	57 (10)		3 (4)	68 (9)		10 (4)	61 (10)		0 (0)	71 (9)		2 (2)	69 (9)	
**Perforation**																		
**No**	82 (92)	756 (98)	0.002	309 (97)	529 (97)	0.723	69 (91)	769 (98)	< 0.001	256 (97)	582 (97)	0.770	34 (89)	804 (98)	0.003	84 (94)	754 (98)	0.086
**Yes**	7 (8)	17 (2)		8 (3)	16 (3)		7 (9)	17 (2)		8 (3)	16 (3)		4 (11)	20 (2)		5 (6)	19 (2)	
**Emergency OP**																		
**No**	84 (94)	744 (96)	0.392	308 (97)	520 (95)	0.204	71 (93)	757 (96)	0.217	256 (97)	572 (96)	0.359	35 (92)	793 (96)	0.201	86 (97)	742 (96)	0.769
**Yes**	5 (6)	29 (4)		9 (3)	25 (5)		5 (7)	29 (4)		8 (3)	26 (4)		3 (8)	31 (4)		3 (3)	31 (4)	
**Adjuvant therapy**																		
**No**	66 (74)	594 (77)	0.571	257 (81)	403 (74)	0.017	56 (74)	604 (77)	0.535	215 (81)	445 (74)	0.025	30 (79)	630 (76)	0.723	70 (79)	590 (76)	0.624
**Yes**	23 (26)	179 (23)		60 (19)	142 (26)		20 (26)	182 (23)		49 (19)	153 (26)		8 (21)	194 (24)		19 (21)	183 (24)	
**PD-L1 TC**																		
**TC ≥ 1%**				53 (17)	36 (7)	< 0.001				38 (14)	38 (6)	< 0.001				12 (13)	26 (3)	< 0.001
**TC < 1%**				264 (83)	509 (93)					226 (86)	560 (94)					77 (87)	747 (97)	
**PD-L1 IC**																		
**IC ≥ 5%**	53 (60)	264 (34)	< 0.001				38 (50)	226 (29)	< 0.001				12 (32)	77 (9)	< 0.001			
**IC < 5%**	36 (40)	509 (66)					38 (50)	560 (71)					26 (68)	747 (91)				

*Abbreviations* CRM, circumferential resection margin; dMMR, deficient mismatch repair; EMVI, extramural vascular invasion; IC, immune cells; MMR, mismatch repair; OP, operation; PD-L1, programmed cell death-ligand 1; pMMR, proficient mismatch repair; PNI, perineural invasion; TC, tumor cells

### Prognostic information of PD-L1 expression in immune cells

**Fig. 2:**
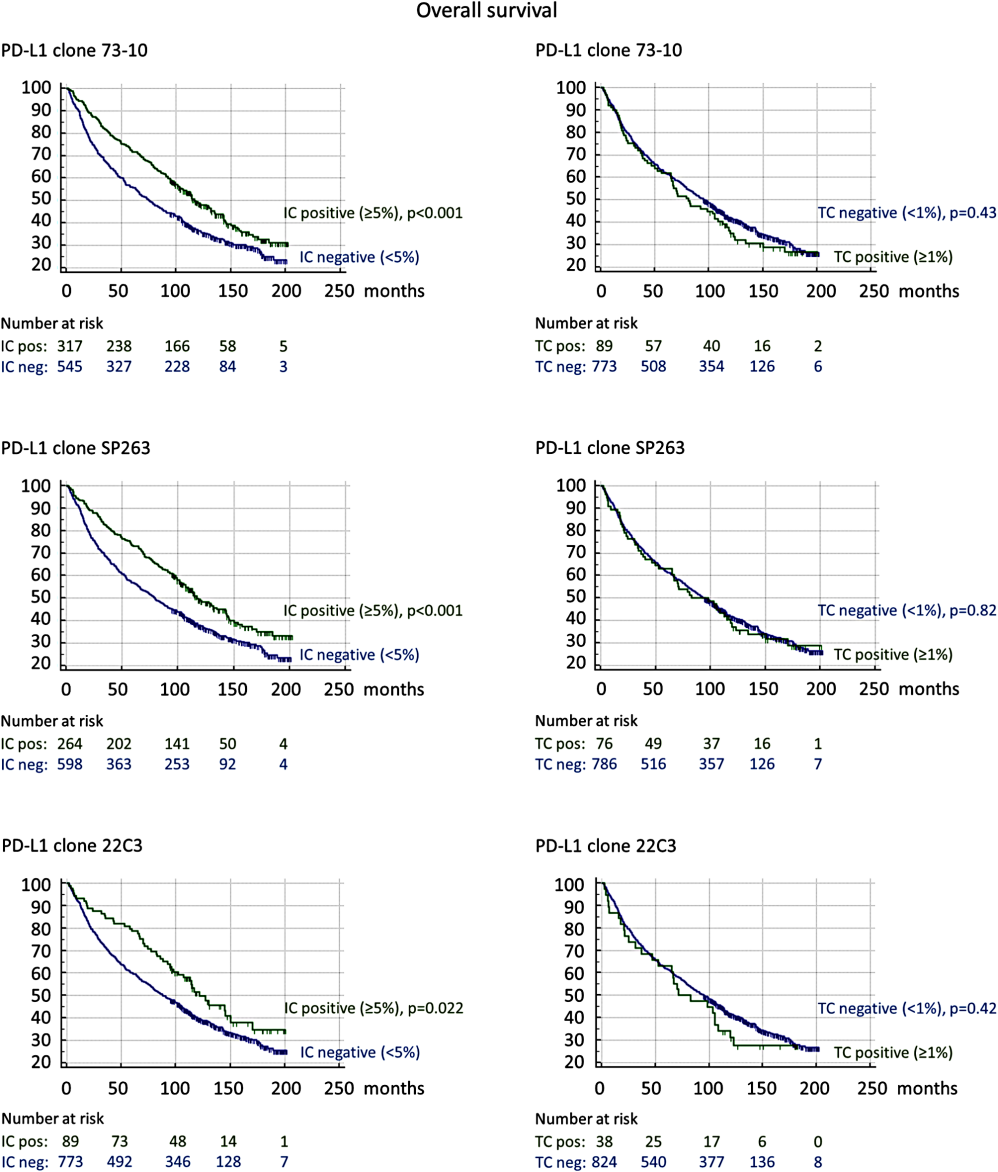
Kaplan-Meier survival curves depicting overall survival (OS) stratified by the expression of programmed death ligand-1 (PD-L1) in tumor cells (TC) and immune cells (IC) for different antibody clones in 862 resected colorectal cancers without neoadjuvant treatment. P-values were calculated by log-rank test

**Fig. 3:**
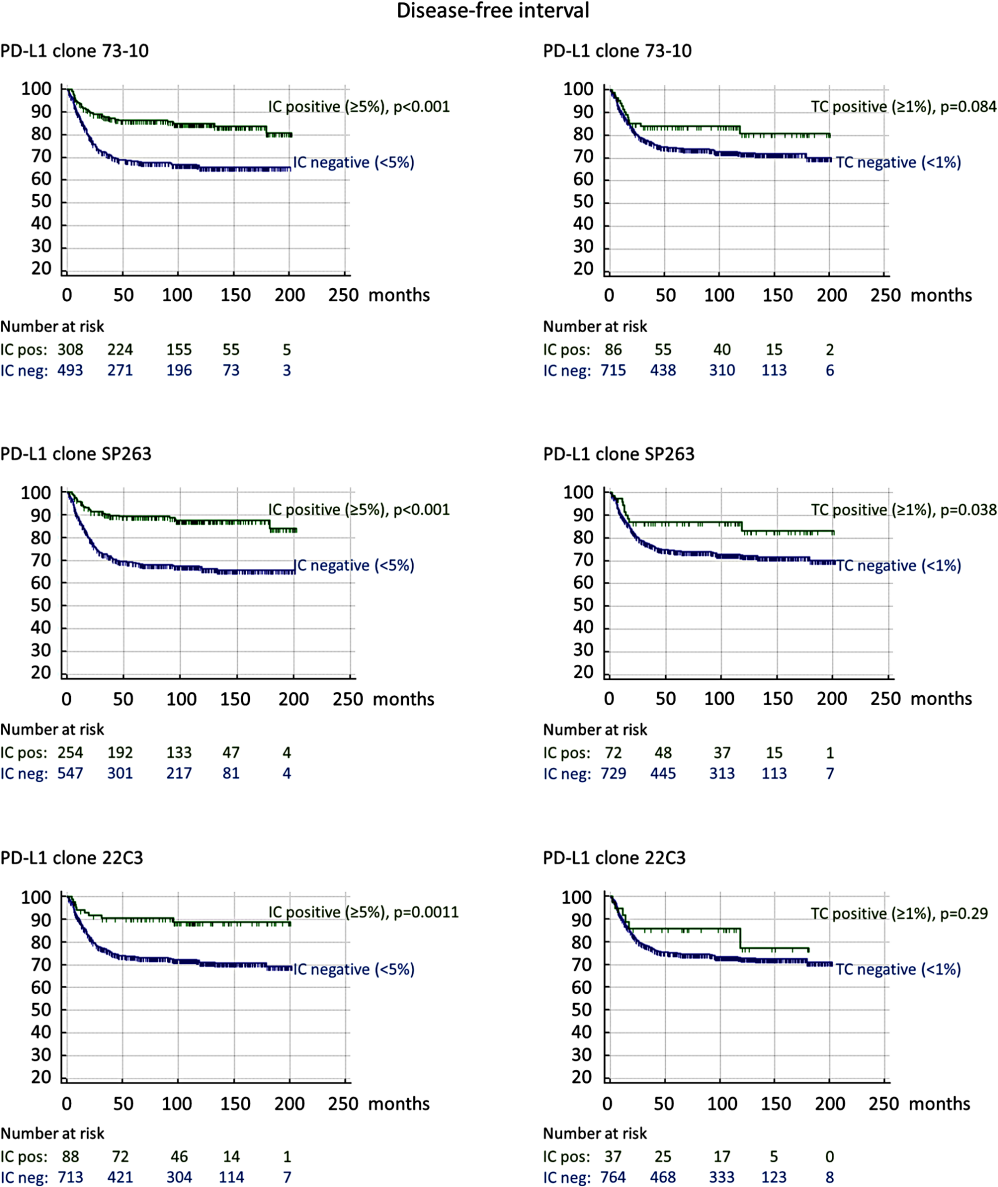
Kaplan-Meier survival curves depicting disease-free interval (DFI) stratified by the expression of programmed death ligand-1 (PD-L1) in tumor cells (TC) and immune cells (IC) for different antibody clones in 801 resected colorectal cancers without neoadjuvant treatment. P-values were calculated by log-rank test

Kaplan-Meier analyses demonstrated that PD-L1 expression in IC was significantly linked to both prolonged OS and DFI for all three antibody clones: 73-10 (both log rank *p* < 0.001), SP263 (both log rank *p* < 0.001), and 22C3 (log rank *p* = 0.022, and log rank *p* = 0.0011, respectively). All Kaplan-Meier curves are shown in Fig. 2 (OS) and Fig. 3 (DFI).

Univariable Cox regression analyses confirmed that PD-L1 expression in IC was associated with both prolonged OS and DFI for all three clones: 73-10; HR = 0.701 (0.587–0.837) and HR = 0.413 (0.296–0.577), SP263; HR = 0.697 (0.577–0.841) and HR = 0.332 (0.225–0.489), and for 22C3; HR = 0.708 (0.527–0.953) and HR = 0.346 (0.177–0.674), respectively (Table 3). The association with prolonged DFI remained significant in multivariable analyses for 73-10 HR = 0.629 (0.443–0.891) and SP263 HR = 0.495 (0.332–0.737), while a trend was seen for 22C3 HR = 0.516 (0.260–1.021), after adjustment for sex, stage, differentiation grade, CRM, MMR, EMVI, PNI, tumor budding, tumor deposit, tumor perforation, emergency operation, and adjuvant treatment (Table 4). Further, the association with prolonged OS remained significant in multivariable analyses for 73-10 HR = 0.798 (0.662–0.961), while a trend was seen for SP263 HR = 0.833 (0.687–1.010), after adjustment for age, sex, stage, differentiation grade, CRM, EMVI, PNI, tumor budding, tumor deposit, perforation, and adjuvant treatment. The association with prolonged OS for 22C3 did not remain significant in the multivariable analysis. The results of the univariable and multivariable Cox regression analyses are shown in Tables 3 and 4, the latter including only results for PD-L1, while full data for the multivariable analyses are found in Supplementary Table 3 (OS) and 4 (DFI).

**Table 3:** Univariable Cox regression proportional hazard models in 862 resected colorectal cancers without neoadjuvant treatment

	Overall survival		Disease-free interval	
	HR (95% CI)	*p*-value	HR (95% CI)	*p*-value
**Age**				
**<74**	1		1	
**≥74**	3.047 (2.523–3.680)	< 0.001	1.161 (0.875–1.542)	0.301
**Sex**				
**Male**	1		1	
**Female**	0.816 (0.690–0.964)	0.017	0.754 (0.570–0.997)	0.048
**Site**				
**Right colon**	1		1	
**Left colon**	0.867 (0.721–1.044	0.132	1.048 (0.768–1.430)	0.769
**Rectum**	0.885 (0.679–1.152)	0.363	1.331 (0.890–1.991)	0.164
**T stage**				
**1**	1		n/a (no events)	
**2**	0.793 (0.444–1.414)	0.432	1	
**3**	1.010 (0.603–1.692)	0.971	2.632 (1.288–5.380)	0.008
**4**	1.991 (1.160–3.416)	0.012	9.125 (4.372–19.043)	< 0.001
**N stage**				
**0**	1		1	
**1**	1.132 (0.921–1.392)	0.240	2.459 (1.721–3.513)	< 0.001
**2**	2.065 (1.679–2.540)	< 0.001	5.742 (4.101–8.039)	< 0.001
**M stage**				
**0**	1		1	
**1**	4.968 (3.906–6.319)	< 0.001	4.535 (2.821–7.291)	< 0.001
**Stage**				
**I**	1		1	
**II**	1.039 (0.778–1.388)	0.794	2.531 (1.089–5.883)	0.031
**III**	1.263 (0.942–1.694)	0.119	7.579 (3.336–17.215)	< 0.001
**IV**	5.551 (3.947–7.807)	< 0.001	18.984 (7.571–47.595)	< 0.001
**Tumor grade**				
**Low grade**	1		1	
**High grade**	1.469 (1.223–1.764)	< 0.001	1.876 (1.402–2.509)	< 0.001
**Mucinous histology**				
**Absent**	1		1	
**Present (> 50%)**	1.006 (0.817–1.238)	0.958	0.857 (0.597–1.231)	0.404
**CRM**				
**>0 mm**	1		1	
**0 mm**	4.940 (3.492–6.991)	< 0.001	6.721 (4.164–10.848)	< 0.001
**MMR**				
**pMMR**	1		1	
**dMMR**	1.096 (0.901–1.335)	0.360	0.423 (0.274–0.654)	< 0.001
**EMVI**				
**No**	1		1	
**Yes**	1.456 (1.229–1.726)	< 0.001	3.696 (2.768–4.934)	< 0.001
**PNI**				
**No**	1		1	
**Yes**	2.058 (1.632–2.596)	< 0.001	3.811 (2.748–5.285)	< 0.001
**Tumor budding**				
**Bd1**	1		1	
**Bd2**	1.427 (1.176–1.730)	< 0.001	2.634 (1.911–3.632)	< 0.001
**Bd3**	1.524 (1.224–1.899)	< 0.001	2.477 (1.711–3.586)	< 0.001
**Tumor deposits**				
**No**	1		1	
**Yes**	2.440 (1.859–3.204)	< 0.001	4.508 (3.135–6.483)	< 0.001
**Perforation**				
**No**	1		1	
**Yes**	1.635 (1.022–2.616)	0.040	3.183 (1.773–5.711)	< 0.001
**Emergency OP**				
**No**	1		1	
**Yes**	1.069 (0.691–1.653)	0.764	1.662 (0.927–2.981)	0.089
**Adjuvant treatment**				
**No**	1		1	
**Yes**	0.522 (0.418–0.653)	< 0.001	1.651 (1.233–2.210)	< 0.001
**PD-L1 73** **-** **10 TC**				
**TC < 1%**	1		1	
**TC ≥ 1%**	1.112 (0.854–1.448)	0.431	0.622 (0.361–1.071)	0.087
**PD-L1 73** **-** **10 IC**				
**IC < 5%**	1		1	
**IC ≥ 5%**	0.701 (0.587–0.837)	< 0.001	0.413 (0.296–0.577)	< 0.001
**PD-L1 SP263 TC**				
**TC < 1%**	1		1	
**TC ≥ 1%**	1.034 (0.775–1.379)	0.820	0.517 (0.273–0.976)	0.042
**PD-L1 SP263 IC**				
**IC < 5%**	1		1	
**IC ≥ 5%**	0.697 (0.577–0.841)	< 0.001	0.332 (0.225–0.489)	< 0.001
**PD-L1 22C3 TC**				
**TC < 1%**	1		1	
**TC ≥ 1%**	1.174 (0.797–1.729)	0.416	0.645 (0.286–1.453)	0.290
**PD-L1 22C3 IC**				
**IC < 5%**	1		1	
**IC ≥ 5%**	0.708 (0.527–0.953)	0.023	0.346 (0.177–0.674)	0.002

*Abbreviations* CRM, circumferential resection margin; dMMR, deficient mismatch repair; EMVI, extramural vascular invasion; IC, immune cells; MMR, mismatch repair; OP, operation; PD-L1, programmed cell death-ligand 1; pMMR, proficient mismatch repair; PNI, perineural invasion; TC, tumor cells

**Table 4:** Results for PD-L1 expression in tumor cells (TC) and immune cells (IC) for different antibody clones in multivariable Cox regression proportional hazard models in 862 resected colorectal cancers without neoadjuvant treatment (full data in supplementary tables 3 and 4)

	Overall survival		Disease-free interval	
	HR (95% CI)	*p*-value	HR (95% CI)	*p*-value
**PD-L1 73**-**10 TC**				
**TC < 1%**	1	0.660	1	0.025
**TC ≥ 1%**	0.937 (0.703–1.250)		0.498 (0.271–0.915)	
**PD-L1 73** **-** **10 IC**				
**IC < 5%**	1	0.018	1	0.010
**IC ≥ 5%**	0.798 (0.662–0.961)		0.629 (0.443–0.891)	
**PD-L1 SP263 TC**				
**TC < 1%**	1	0.633	1	0.050
**TC ≥ 1%**	0.927 (0.680–1.264)		0.487 (0.238–0.997)	
**PD-L1 SP263 IC**				
**IC < 5%**	1	0.064	1	< 0.001
**IC ≥ 5%**	0.833 (0.687–1.010)		0.495 (0.332–0.737)	
**PD-L1 22C3 TC**				
**TC < 1%**	1	0.438	1	0.312
**TC ≥ 1%**	0.847 (0.557–1.287)		0.618 (0.244–1.564)	
**PD-L1 22C3 IC**				
**IC < 5%**	1	0.241	1	0.059
**IC ≥ 5%**	0.835 (0.618–1.128)		0.516 (0.260–1.021)	

*Abbreviations* HR, hazard ratio; IC, immune cells; PD-L1, programmed cell death-ligand 1; TC, tumor cells

### Prognostic information of PD-L1 expression in tumor cells

Kaplan-Meier analyses demonstrated that PD-L1 expression in TC was significantly linked to prolonged DFI regarding SP263 (log rank *p* = 0.038), while a trend was seen for 73-10 (log rank *p* = 0.084), but no association was seen for 22C3. PD-L1 expression in TC did not show a significant prognostic value regarding OS for any antibody clone. All Kaplan-Meier curves are shown in Fig. 2 (OS) and Fig. 3 (DFI).

Univariable Cox regression analyses confirmed that PD-L1 expression in TC was associated with prolonged DFI regarding SP263 HR = 0.517 (0.273–0.976), while a trend was seen for 73-10 HR = 0.622 (0.361–1.071). No association was seen for 22C3 (Table 3). The association with prolonged DFI remained significant for SP263 HR = 0.487 (0.238–0.997) and was strengthened for 73-10 HR = 0.498 (0.271–0.915) in multivariable analyses adjusted as for IC above (Table 4). PD-L1 expression in TC was not prognostic of OS for any clone in either univariable or multivariable Cox regression analyses. The results of the univariable and multivariable Cox regression analyses are shown in Tables 3 and 4, respectively. Full data for the multivariable analyses are found in Supplementary Table 3 (OS) and 4 (DFI).

## Discussion

In this countywide population-based cohort of surgically treated CRC without prior neoadjuvant therapy, we found that the PD-L1 positivity rate and the prognostic impact of PD-L1 expression in IC and TC differed between antibody clones. PD-L1 expression in IC was an independent prognostic factor for prolonged DFI regarding clones 73-10 and SP263 (e.g. providing more prognostic information than tumor budding, see Supplementary Table 4), while a non-significant trend was seen for 22C3. For OS, the equivalent could only be shown for 73-10. PD-L1 expression in TC was an independent prognostic factor for prolonged DFI for 73-10 and SP263. However, PD-L1 expression in TC was not prognostic of OS for any of the clones.

PD-L1 expression in tumor cells is associated with poor prognosis in several malignancies [14–16], while partly contradictory and inconclusive results have been presented for CRC. PD-L1 expression has been associated with a better prognosis in some studies [10, 23, 25], and with a worse prognosis in others [17–21, 26]. The lack of a standardized approach to assess PD-L1 in CRC may explain part of the differences in results. Different studies have used different cut-offs to define PD-L1 positivity. In our material, we used 1% TC and 5% IC as cut-offs, based on differences in survival analyses and on data from some previous studies with similar designs to ours [18, 20, 27, 28]. In addition, some studies evaluate only the percentage of stained cells, while others combine the percentage of stained cells and the degree of staining intensity, resulting in a variety of results that are scarcely comparable. The variations in methodology are also reflected in the differences in the reported prevalence of PD-L1 positivity in CRC, ranging from 5% [18] to 89% [22] in TC and from 5% [22] to 61% [27] in IC. In the present study, PD-L1 expression in TC was defined as any cell membrane staining, partial or complete, regardless of intensity, while PD-L1 expression in IC was defined as any cell membrane staining or cytoplasmatic staining, regardless of intensity, which is consistent with the FDA-approved PD-L1 IHC staining protocols [29].

An important factor for a cohort spanning many years is the possible confounding introduced by tissue degradation in stored tumor specimens. Fading of PD-L1 expression in FFPE specimens depending on the age of the tumor blocks has been previously described in studies on lung cancer [30, 31]. This was especially significant when samples were older than 3 years. However, the decrease in PD-L1 expression was essentially limited to TC while IC showed a similar PD-L1 expression [31]. In our study, no obvious differences or tendencies to fading could be demonstrated for any antibody clone in either TC or IC.

Several studies have investigated differences in expression between different antibody clones of PD-L1 in solid tumors. In two comprehensive studies regarding PD-L1 expression in lung cancer and urothelial cancer, respectively, three of four assays (22C3, SP263, and 28-8, but not SP142) showed interchangeable results [32, 33]. However, in another study on head and neck squamous cell carcinoma, the differences meant that the eligibility for certain checkpoint inhibitor regimens was highly dependent on the choice of the antibody clone [34]. In our study, regardless of whether TC or IC was examined, the concordance between 73-10 and SP263 was relatively high (but not perfect), while 22C3 identified significantly fewer cases as PD-L1 positive, less than half as many for both TC and IC. 73-10 and SP263 together identified all cases with TC PD-L1 expression and all but three cases (> 99.5%) with IC PD-L1 expression.

To the best of our knowledge, there are few studies that have compared different PD-L1 antibody clones in CRC. Lee et al. [20] compared three different clones (MIH1, E1L3N, and 22C3) in 336 resected CRC cases. Cut-offs and study design were similar to ours, and positivity rates differed between clones. The degree of correlation and concordance between E1L3N and 22C3 were relatively high, whereas M1H1 was considered an outlier. Multivariable analyses indicated that PD-L1 positivity for 22C3 in TC and its negativity in IC were independent predictors of impaired OS and DFS. MIH1 correlated to OS and E1L3N to OS and DFS only in univariable analyses. Another study, by Lang-Schwarz et al. [28], compared two different antibody clones of PD-L1 (22C3 and QR1). While more cases were positive for clone QR1 than 22C3 in that study, PD-L1 expression in IC (and in IC and/or TC) was linked to favourable OS for both clones.

In our material, PD-L1 expression in IC was significantly associated with several favourable clinicopathological features like lower T stage, lower N stage, no distant metastasis (M0), lower total stage, absence of EMVI, low extent of tumor budding, and absence of tumor deposits for all three clones. PD-L1 expression in IC was also associated with the absence of PNI for 73-10 and SP263, and with low tumor grade for clone 73-10 only. Unsurprisingly, PD-L1 expression in IC was linked to both prolonged OS and DFI for all three clones. The link to prolonged DFI remained significant in multivariable analyses for 73-10 and SP263, and to OS for 73-10. Wyss et al. [27] found similar results in their study on 279 patients with CRC using clone SP142, where stromal PD-L1 expression was associated with less aggressive tumor behaviour and better OS and DFS. Several other studies have also reported analogous results [20, 28, 35].

In the present study, PD-L1 expression in TC was significantly associated with high tumor grade and the presence of tumor perforation for all three clones. None of the other clinicopathological parameters associated with poor outcome showed any relation in this regard, which, however, several other studies have shown. Rosenbaum et al. [26] and Wyss et al. [27] showed a correlation between TC PD-L1 expression and poorly differentiated tumors in their studies. The latter study additionally showed a correlation to lymph node metastasis, while Secinti et al. [36] showed a correlation to distant metastases, PNI, and a high degree of tumor budding. Given the association with unfavourable clinicopathological features, it is somewhat unexpected in our study that PD-L1 expression in TC was linked to prolonged DFI (but not OS) for SP263 and 73-10. These results are contradictory to other studies that have rather shown a correlation between PD-L1 expression in TC and significantly worse DFS and OS [17–21, 26, 36]. However, some studies have shown the opposite, thus supporting our findings. Droeser et al. [10] showed that expression of PD-L1 in TC in pMMR CRC was significantly associated with lower T stage, absence of lymph node metastases, low grade, absence of vascular invasion, and significantly improved survival in terms of OS. Similar correlations to favourable clinicopathological features were presented by Al-Jussani et al. [25]. However, the latter used Combined Positive Score (CPS) in the assessment of PD-L1 expression, so the results are not perfectly comparable. Li et al. [23] showed that expression of PD-L1 in TC was associated with prolonged OS and DFS in Kaplan-Meier analyses. However, the associations were not maintained in multivariable analyses. Most interestingly, Wyss et al. [27] showed a trend towards prolonged DFS despite the above-described associations with unfavourable clinicopathological factors. Patients with tumor PD-L1 expression had a 100% 5-year DFS compared to 77% for patients without PD-L1 positivity, according to their findings.

A limitation of our study is that the assessment of PD-L1 was performed on TMAs. As PD-L1 expression may be focal or heterogeneous, the proportion of positive cases may be falsely underrepresented. To reduce the risk of bias from intratumoral heterogeneity, two cores per tumor were assessed. However, in a comparison between different antibodies or different antibody clones, as in the present study, TMAs are excellent as the same area is evaluated in a large number of cases. Furthermore, all stainings were evaluated by a single senior pathologist only, which can also be seen as a strength, as it better reflects the clinical reality. Finally, we did not have complete data on clinically relevant prognostic information, e.g. pre-operative serum carcinoembryonic antigen (CEA).

## Conclusions

In surgically treated CRC, the prevalence of positivity and prognostic impact of PD-L1 expression in both IC and TC differ between antibody clones. PD-L1 expression in IC was associated with prolonged OS and DFI in univariable analysis for all three clones, and the associations remained in multivariable analyses for 73-10. Our results suggest the potential use of PD-L1 as a prognostic marker and that 73-10 and SP263 are the best candidates for further studies, which are needed in order to standardize PD-L1 assessment in CRC.

### Electronic supplementary material

Below is the link to the electronic supplementary material.

**Supplementary Material 1: Supplementary table 1.** Prevalence of immunohistochemical PD-L1 positivity in tumor cells (TC) and immune cells (IC) depending on antibody clone and cut-off in 862 cases of non-neoadjuvant treated resected colorectal cancer. (DOCX 17 kB)

**Supplementary Material 2: Supplementary table 2.** PD-L1 expression in tumor cells (TC) and immune cells (IC) depending on antibody clone and age of tissue blocks in 862 cases of colorectal cancer from 2007 to 2015. (DOCX 17 kB)

**Supplementary Material 3: Supplementary table 3.** Multivariable Cox regression analyses investigating the link to overall survival (OS) for immunohistochemical PD-L1 positivity depending on antibody clone and cell type in 862 cases of non-neoadjuvant treated resected colorectal cancer. (DOCX 27 kB)

**Supplementary Material 4: Supplementary table 4.** Multivariable Cox regression analyses investigating the link to disease-free interval (DFI) for immunohistochemical PD-L1 positivity depending on antibody clone and cell type in 801 cases of non-neoadjuvant treated resected colorectal cancer. (DOCX 29 kB)

**Supplementary Material 5: Supplementary Fig. 1.** Flowchart of the patients included in the study

## Data Availability

Availability of data and materials: The datasets used and/or analysed during the current study are available from the corresponding author on reasonable request.

## Abbreviations

CRC: Colorectal cancer
CEA: Carcinoembryonic antigen
CRM: Circumferential resection margin
CTLA-4: Cytotoxic T-lymphocyte associated protein 4
DFI: Disease-free interval
DFS: Disease-free survival
dMMR: Deficient mismatch repair
EMVI: Extramural vascular invasion
FFPE: Formalin-fixed paraffin-embedded
HPA: The Human Protein Atlas
HR: Hazard ratio
IC: Immune cells
ICI: Immune checkpoint inhibition
IHC: Immunohistochemistry
MSI: Microsatellite instability
OS: Overall survival
PD-1: Programmed death receptor-1
PD-L1: Programmed cell death-ligand 1
pMMR: Proficient mismatch repair
PNI: Perineural invasion
PTL: Primary tumor location
TC: Tumor cells
TCGA: The Cancer Genome Atlas
TILs: Tumor-infiltrating lymphocytes
TMA: Tissue microarray
