# Supplementary material for: The prognostic value of programmed death-ligand 1 (PD-L1) expression in resected colorectal cancer without neoadjuvant therapy - differences between antibody clones and cell types
Source: BMC Cancer. 2024 Aug 26;24:1051. doi: 10.1186/s12885-024-12812-7 (PMC11346183; doi:10.1186/s12885-024-12812-7)
Supplement: Supplementary file 1 — Supplementary Material 1: Supplementary table 1. Prevalence of immunohistochemical PD-L1 positivity in tumor cells (TC) and immune cells (IC) depending on antibody clone and cut-off in 862 cases of non-neoadjuvant treated resected colorectal cancer. (DOCX 17 kB) [file 12885_2024_12812_MOESM1_ESM.docx]

**Supplementary Table 1**. Prevalence of immunohistochemical PD-L1 positivity in tumor cells (TC) and immune cells (IC) depending on antibody clone and cut-off in 862 cases of non-neoadjuvant treated resected colorectal cancer.

|  | **TC, n (%)** | **IC, n (%)** |
| --- | --- | --- |
| **73-10** |  |  |
| **≥ 1%** | 89 (10.3) | 602 (69.8) |
| **≥ 5%** | 55 (6.4) | 317 (36.8) |
| **≥ 10%** | 42 (4.9) | 125 (14.5) |
| **≥ 50%** | 27 (3.1) | 6 (0.7) |
| **SP263** |  |  |
| **≥ 1%** | 76 (9.2) | 578 (67.1) |
| **≥ 5%** | 47 (5.5) | 264 (30.6) |
| **≥ 10%** | 33 (3.8) | 99 (11.5) |
| **≥ 50%** | 22 (2.6) | 2 (0.2) |
| **22C3** |  |  |
| **≥ 1%** | 38 (4.4) | 357 (41.4) |
| **≥ 5%** | 29 (3.4) | 89 (10.3) |
| **≥ 10%** | 19 (2.2) | 14 (1.6) |
| **≥ 50%** | 7 (0.8) | 0 (0) |
