# Supplementary material for: The prognostic value of programmed death-ligand 1 (PD-L1) expression in resected colorectal cancer without neoadjuvant therapy - differences between antibody clones and cell types
Source: BMC Cancer. 2024 Aug 26;24:1051. doi: 10.1186/s12885-024-12812-7 (PMC11346183; doi:10.1186/s12885-024-12812-7)
Supplement: Supplementary file 2 — Supplementary Material 2: Supplementary table 2. PD-L1 expression in tumor cells (TC) and immune cells (IC) depending on antibody clone and age of tissue blocks in 862 cases of colorectal cancer from 2007 to 2015. (DOCX 17 kB) [file 12885_2024_12812_MOESM2_ESM.docx]

**Supplementary Table 2**. PD-L1 expression in tumor cells (TC) and immune cells (IC) depending on antibody clone and age of tissue blocks in 862 cases of colorectal cancer from 2007-2015.

| **Year of tissue blocks** | **73-10, n (%)** | | **SP263, n (%)** | | **22C3, n (%)** | |
| --- | --- | --- | --- | --- | --- | --- |
|  | **TC ≥1%** | **IC ≥5%** | **TC ≥1%** | **IC ≥5%** | **TC ≥1%** | **IC ≥5%** |
| **2007-2009**  **(n=301)** | 44  (15) | 95  (32) | 38  (13) | 84  (28) | 21  (7) | 27  (9) |
| **2010-2012**  **(n=268)** | 19  (7) | 94  (35) | 19  (7) | 78  (29) | 9  (3) | 26  (10) |
| **2013-2015**  **(n=293)** | 26  (9) | 128  (44) | 19  (6) | 102  (35) | 8  (3) | 36  (12) |
