# Supplementary material for: The prognostic value of programmed death-ligand 1 (PD-L1) expression in resected colorectal cancer without neoadjuvant therapy - differences between antibody clones and cell types
Source: BMC Cancer. 2024 Aug 26;24:1051. doi: 10.1186/s12885-024-12812-7 (PMC11346183; doi:10.1186/s12885-024-12812-7)
Supplement: Supplementary file 3 — Supplementary Material 3: Supplementary table 3. Multivariable Cox regression analyses investigating the link to overall survival (OS) for immunohistochemical PD-L1 positivity depending on antibody clone and cell type in 862 cases of non-neoadjuvant treated resected colorectal cancer. (DOCX 27 kB) [file 12885_2024_12812_MOESM3_ESM.docx]

**Supplementary Table 3**. Multivariable Cox regression analyses investigating the link to overall survival (OS) for immunohistochemical PD-L1 positivity depending on antibody clone and cell type in 862 cases of non-neoadjuvant treated resected colorectal cancer.

|  | **TC** (<1%) | | | **IC** (<5%) | | |
| --- | --- | --- | --- | --- | --- | --- |
| **Characteristics** (reference value) | **p-value** | **HR** | **CI** | **p-value** | **HR** | **CI** |
| **Age** (<74) | <0.001 | 2.643 | 2.168-3.222 | <0.001 | 2.635 | 2.163-3.211 |
| **Sex** (male) | 0.170 | 0.888 | 0.750-1.051 | 0.216 | 0.899 | 0.760-1.063 |
| **Stage** (I) | <0.001 | 1.743 | 1.515-2.006 | <0.001 | 1.710 | 1.484-1.970 |
| **Tumor grade** (low grade) | 0.177 | 1.151 | 0.939-1.410 | 0.099 | 1.178 | 0.971-1.428 |
| **CRM** (>0) | <0.001 | 3.318 | 2.299-4.789 | <0.001 | 3.225 | 2.236-4.652 |
| **EMVI** (no) | 0.101 | 1.181 | 0.969-1.439 | 0.115 | 1.172 | 0.963-1.426 |
| **PNI** (no) | <0.001 | 1.599 | 1.236-2.069 | <0.001 | 1.556 | 1.203-2.013 |
| **Tumor budding** (Bd1) | 0.036 | 1.128 | 1.008-1.261 | 0.069 | 1.110 | 0.993-1.241 |
| **Tumor deposit** (no) | <0.001 | 1.715 | 1.279-2.299 | <0.001 | 1.750 | 1.306-2.346 |
| **Perforation** (no) | 0.124 | 1.466 | 0.903-2.380 | 0.125 | 1.460 | 0.902-2.362 |
| **Adjuvant treatment** (no) | <0.001 | 0.361 | 0.280-0.465 | <0.001 | 0.357 | 0.278-0.460 |
| **PD-L1 73-10** (TC <1% / IC <5%) | 0.660 | 0.937 | 0.703-1.250 | 0.018 | 0.798 | 0.662-0.961 |
|  |  |  |  |  |  |  |
| **Age** (<74) | <0.001 | 2.647 | 2.171-3.227 | <0.001 | 2.633 | 2.161-3.209 |
| **Sex** (male) | 0.175 | 0.889 | 0.750-1.053 | 0.162 | 0.887 | 0.750-1.049 |
| **Stage** (I) | <0.001 | 1.744 | 1.515-2.007 | <0.001 | 1.718 | 1.491-1.979 |
| **Tumor grade** (low grade) | 0.174 | 1.149 | 0.942-1.402 | 0.173 | 1.142 | 0.944-1.382 |
| **CRM** (>0) | <0.001 | 3.318 | 2.299-4.789 | <0.001 | 3.299 | 2.289-4.755 |
| **EMVI** (no) | 0.104 | 1.179 | 0.968-1.437 | 0.104 | 1.178 | 0.968-1.434 |
| **PNI** (no) | <0.001 | 1.602 | 1.239-2.072 | <0.001 | 1.570 | 1.214-2.030 |
| **Tumor budding** (Bd1) | 0.037 | 1.127 | 1.008-1.261 | 0.060 | 1.114 | 0.996-1.246 |
| **Tumor deposit** (no) | <0.001 | 1.709 | 1.274-2.292 | <0.001 | 1.731 | 1.292-2.320 |
| **Perforation** (no) | 0.122 | 1.471 | 0.905-2.390 | 0.119 | 1.469 | 0.908-2.377 |
| **Adjuvant treatment** (no) | <0.001 | 0.362 | 0.281-0.465 | <0.001 | 0.361 | 0.280-0.465 |
| **PD-L1 SP263** (TC <1% / IC <5%) | 0.633 | 0.927 | 0.680-1.264 | 0.064 | 0.833 | 0.687-1.010 |
|  |  |  |  |  |  |  |
| **Age** (<74) | <0.001 | 2.651 | 2.175-3.231 | <0.001 | 2.651 | 2.175-3.231 |
| **Sex** (male) | 0.185 | 0.892 | 0.753-1.056 | 0.176 | 0.890 | 0.753-1.053 |
| **Stage** (I) | <0.001 | 1.748 | 1.519-2.012 | <0.001 | 1.731 | 1.503-1.993 |
| **Tumor grade** (low grade) | 0.142 | 1.163 | 0.952-1.421 | 0.180 | 1.140 | 0.942-1.379 |
| **CRM** (>0) | <0.001 | 3.325 | 2.305-4.797 | <0.001 | 3.357 | 2.327-4.842 |
| **EMVI** (no) | 0.118 | 1.173 | 0.961-1.430 | 0.117 | 1.172 | 0.962-1.428 |
| **PNI** (no) | <0.001 | 1.609 | 1.244-2.081 | <0.001 | 1.599 | 1.237-2.067 |
| **Tumor budding** (Bd1) | 0.041 | 1.125 | 1.006-1.258 | 0.042 | 1.124 | 1.005-1.257 |
| **Tumor deposit** (no) | <0.001 | 1.702 | 1.269-2.283 | <0.001 | 1.718 | 1.282-2.301 |
| **Perforation** (no) | 0.113 | 1.483 | 0.914-2.407 | 0.122 | 1.465 | 0.905-2.370 |
| **Adjuvant treatment** (no) | <0.001 | 0.360 | 0.280-0.464 | <0.001 | 0.363 | 0.282-0.467 |
| **PD-L1 22C3** (TC <1% / IC <5%) | 0.438 | 0.847 | 0.557-1.287 | 0.241 | 0.835 | 0.618-1.128 |

Abbreviations: CRM, circumferential resection margin; EMVI, extramural vascular invasion; IC, immune cells; PD-L1, programmed cell death-ligand 1; PNI, perineural invasion; TC, tumor cells.
