# Supplementary material for: The prognostic value of programmed death-ligand 1 (PD-L1) expression in resected colorectal cancer without neoadjuvant therapy - differences between antibody clones and cell types
Source: BMC Cancer. 2024 Aug 26;24:1051. doi: 10.1186/s12885-024-12812-7 (PMC11346183; doi:10.1186/s12885-024-12812-7)
Supplement: Supplementary file 4 — Supplementary Material 4: Supplementary table 4. Multivariable Cox regression analyses investigating the link to disease-free interval (DFI) for immunohistochemical PD-L1 positivity depending on antibody clone and cell type in 801 cases of non-neoadjuvant treated resected colorectal cancer. (DOCX 29 kB) [file 12885_2024_12812_MOESM4_ESM.docx]

**Supplementary Table 4**. Multivariable Cox regression analyses investigating the link to disease-free interval (DFI) for immunohistochemical PD-L1 positivity depending on antibody clone and cell type in 801 cases of non-neoadjuvant treated resected colorectal cancer.

|  | **TC** (<1%) | | | **IC** (<5%) | | |
| --- | --- | --- | --- | --- | --- | --- |
| **Characteristics** (reference value) | **p-value** | **HR** | **CI** | **p-value** | **HR** | **CI** |
| **Sex** (male) | 0.243 | 0.842 | 0.632-1.122 | 0.311 | 0.861 | 0.645-1.149 |
| **Stage** (I) | <0.001 | 2.159 | 1.650-2.825 | <0.001 | 2.099 | 1.602-2.751 |
| **Tumor grade** (low grade) | 0.005 | 1.606 | 1.156-2.231 | 0.013 | 1.515 | 1.094-2.100 |
| **CRM** (>0) | <0.001 | 4.318 | 2.575-7.243 | <0.001 | 3.772 | 2.273-6.259 |
| **MMR** (pMMR) | 0.011 | 0.520 | 0.316-0.856 | 0.007 | 0.504 | 0.307-0.825 |
| **EMVI** (no) | <0.001 | 1.909 | 1.365-2.671 | <0.001 | 1.890 | 1.355-2.636 |
| **PNI** (no) | 0.019 | 1.577 | 1.081-2.301 | 0.017 | 1.579 | 1.087-2.294 |
| **Tumor budding** (Bd1) | 0.304 | 1.109 | 0.912-1.348 | 0.408 | 1.085 | 0.895-1.315 |
| **Tumor deposit** (no) | <0.001 | 2.076 | 1.395-3.088 | <0.001 | 2.073 | 1.396-3.077 |
| **Perforation** (no) | 0.004 | 2.893 | 1.404-5.960 | 0.012 | 2.549 | 1.230-5.282 |
| **Emergency operation** (no) | 0.461 | 0.769 | 0.384-1.540 | 0.376 | 0.725 | 0.358-1.471 |
| **Adjuvant treatment** (no) | <0.001 | 0.517 | 0.367-0.727 | <0.001 | 0.516 | 0.367-0.725 |
| **PD-L1 73-10** (TC <1% / IC <5%) | 0.025 | 0.498 | 0.271-0.915 | 0.010 | 0.629 | 0.443-0.891 |
|  |  |  |  |  |  |  |
| **Sex** (male) | 0.285 | 0.854 | 0.640-1.139 | 0.272 | 0.851 | 0.638-1.134 |
| **Stage** (I) | <0.001 | 2.143 | 1.634-2.810 | <0.001 | 2.051 | 1.566-2.688 |
| **Tumor grade** (low grade) | 0.008 | 1.559 | 1.124-2.162 | 0.026 | 1.452 | 1.048-2.011 |
| **CRM** (>0) | <0.001 | 4.160 | 2.493-6.942 | <0.001 | 4.016 | 2.425-6.652 |
| **MMR** (pMMR) | 0.012 | 0.524 | 0.317-0.866 | 0.006 | 0.500 | 0.307-0.815 |
| **EMVI** (no) | <0.001 | 1.875 | 1.339-2.625 | <0.001 | 1.905 | 1.367-2.656 |
| **PNI** (no) | 0.011 | 1.633 | 1.121-2.378 | 0.023 | 1.543 | 1.063-2.241 |
| **Tumor budding** (Bd1) | 0.350 | 1.098 | 0.904-1.334 | 0.471 | 1.074 | 0.886-1.301 |
| **Tumor deposit** (no) | <0.001 | 2.015 | 1.355-2.997 | <0.001 | 2.110 | 1.422-3.130 |
| **Perforation** (no) | 0.004 | 2.905 | 1.406-6.002 | 0.009 | 2.618 | 1.276-5.371 |
| **Emergency operation** (no) | 0.468 | 0.772 | 0.385-1.548 | 0.365 | 0.722 | 0.357-1.457 |
| **Adjuvant treatment** (no) | <0.001 | 0.534 | 0.379-0.753 | <0.001 | 0.524 | 0.372-0.736 |
| **PD-L1 SP263** (TC <1% / IC <5%) | 0.050 | 0.487 | 0.238-0.997 | <0.001 | 0.495 | 0.332-0.737 |
|  |  |  |  |  |  |  |
| **Sex** (male) | 0.255 | 0.845 | 0.633-1.128 | 0.267 | 0.845 | 0.636-1.132 |
| **Stage** (I) | <0.001 | 2.218 | 1.696-2.901 | <0.001 | 2.162 | 1.653-2.827 |
| **Tumor grade** (low grade) | 0.015 | 1.502 | 1.083-2.083 | 0.022 | 1.466 | 1.059-2.031 |
| **CRM** (>0) | <0.001 | 3.952 | 2.372-6.586 | <0.001 | 3.989 | 2.400-6.630 |
| **MMR** (pMMR) | 0.006 | 0.487 | 0.293-0.809 | 0.003 | 0.478 | 0.293-0.780 |
| **EMVI** (no) | <0.001 | 1.858 | 1.328-2.600 | <0.001 | 1.850 | 1.326-2.583 |
| **PNI** (no) | 0.009 | 1.658 | 1.137-2.416 | 0.011 | 1.633 | 1.123-2.375 |
| **Tumor budding** (Bd1) | 0.449 | 1.079 | 0.888-1.310 | 0.413 | 1.085 | 0.894-1.316 |
| **Tumor deposit** (no) | <0.001 | 2.076 | 1.398-3.082 | <0.001 | 2.121 | 1.431-3.144 |
| **Perforation** (no) | 0.007 | 2.708 | 1.313-5.588 | 0.008 | 2.717 | 1.310-5.638 |
| **Emergency operation** (no) | 0.495 | 0.784 | 0.391-1.572 | 0.383 | 0.728 | 0.357-1.482 |
| **Adjuvant treatment** (no) | <0.001 | 0.510 | 0.363-0.716 | <0.001 | 0.513 | 0.365-0.720 |
| **PD-L1 22C3** (TC <1% / IC <5%) | 0.312 | 0.618 | 0.244-1.564 | 0.059 | 0.516 | 0.260-1.021 |

Abbreviations: CRM, circumferential resection margin; EMVI, extramural vascular invasion; IC, immune cells; MMR, mismatch repair; PD-L1, programmed cell death-ligand 1; pMMR, proficient mismatch repair; PNI, perineural invasion; TC, tumor cells.
