# Supplementary figures and images for: The prognostic value of programmed death-ligand 1 (PD-L1) expression in resected colorectal cancer without neoadjuvant therapy - differences between antibody clones and cell types
Source: BMC Cancer. 2024 Aug 26;24:1051. doi: 10.1186/s12885-024-12812-7 (PMC11346183; doi:10.1186/s12885-024-12812-7)

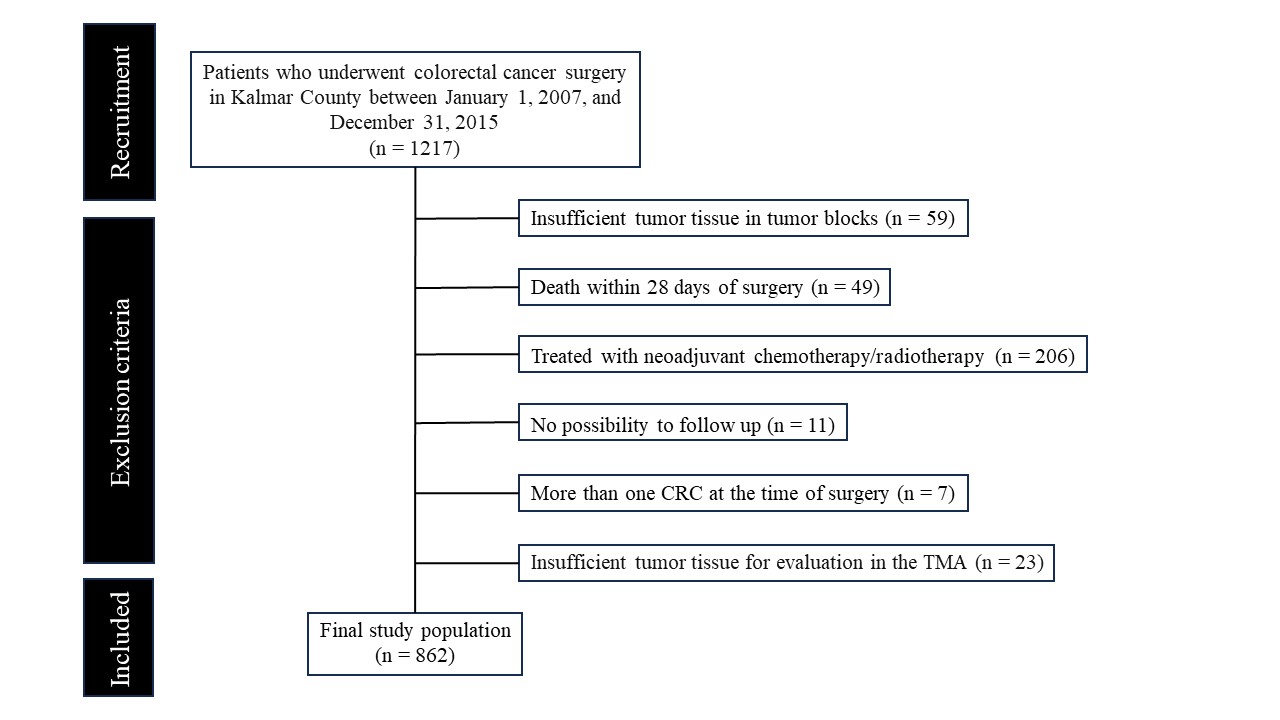

Supplement: Supplementary file 5 — Supplementary Material 5: Supplementary Fig. 1. Flowchart of the patients included in the study [file 12885_2024_12812_MOESM5_ESM.jpg]
